# Positioning and Utilization of Information and Communication Technology in Community Pharmacies of Selangor, Malaysia: Cross-Sectional Study

**DOI:** 10.2196/17982

**Published:** 2020-07-08

**Authors:** Bhuvan KC, Dorothy Lim, Chia Chia Low, Connie Chew, Ali Qais Blebil, Juman Abdulelah Dujaili, Alian A Alrasheedy

**Affiliations:** 1 School of Pharmacy Monash University Malaysia Bandar Sunway Malaysia; 2 Unaizah College of Pharmacy Qassim University Unaizah Saudi Arabia; 3 Department of Pharmacy Practice College of Pharmacy Qassim University Buraydah Saudi Arabia

**Keywords:** information and communication technology, community pharmacy, Malaysia, pharmacy services

## Abstract

**Background:**

Information and communication technology (ICT) is an essential element of modern “smart” cities. These smart cities have integrated housing, marketplace, public amenities, services, business, and transportation via ICT. ICT is also now widely used in urban health care delivery.

**Objective:**

The aim of this study was to determine the positioning and roles of ICT in community pharmacies in the state of Selangor, Malaysia.

**Methods:**

A cross-sectional study was conducted from November 2018 to January 2019 across 9 different subdistricts in the state of Selangor, including Subang Jaya, Cheras, Puchong, Port Klang, Kota Kemuning, Selayang, Chow Kit, Ampang, and Seri Kembangan. A total of 90 community pharmacists were approached from the 9 subdistricts and invited to participate in the study.

**Results:**

Of the 90 community pharmacies approached, 60 agreed to participate in the study, representing a response rate of 67%. The majority (36/60, 60%) of the respondents were women, and more than half (32/60, 53%) of the community pharmacies were run by young adults (ie, 30 years old and younger). More than three-quarters of the community pharmacies (46/60, 77%) used electronic health records. Half of the community pharmacies used online social media platforms for advertising and promoting their pharmacies. The vast majority of the community pharmacies (55/60, 92%) were using modern electronic payment systems, and some were also using other new electronic payment methods. Moreover, most of the community pharmacies (41/60, 68%) were using software and programs for accounting and logistics purposes. In addition, 47/60 (78%) of the community pharmacies used a barcode reading system for medicines/health products, and 16/60 (27%) of the pharmacies had online stores, and consumers could buy medicines and health products from these pharmacies via their online portal. In addition, 20/60 (33%) of the community pharmacies used at least one of the common online business platforms available in Southeast Asia to sell products/medicines. The telephone was the most commonly used means of communication with patients, although some pharmacies also used email, WhatsApp, SMS text messaging, and other communication platforms.

**Conclusions:**

This study showed that the majority of community pharmacies in Selangor, Malaysia are using ICT for different purposes. However, there is still limited use of mobile apps to provide health services. Overall, community pharmacies have been adopting ICT apps for pharmacy services but the rate of adoption is relatively slower than that in other sectors of Malaysia.

## Introduction

Information and communication technology (ICT) is an important element in contemporary business and lifestyle. ICT includes various electronic apps, tools, and services that promote the sharing of information and facilitate communication [1]. Malaysia is undergoing rapid urbanization, with ICT playing an integral role, especially in “smart” cities [2]. Under the concept of smart cities, in which cities are at the interface of the social, economic, and technological dimensions, ICT is used to enhance the quality of life of the population, improve accessibility to services, and ensure consistent improvement in the economy and in sustainable social and environmental developments [3]. ICT apps are used in various activities such as accounting, marketing, staffing, record keeping, manufacturing processes (consumer and industrial goods), and in the service sectors (eg, transportation, education, health care) [2].

The advancement of ICT apps accompanied by reduction in costs and increased availability have resulted in their increased use and improvement along with related services for various functions by both the public and private sectors. The health care sector has also increasingly adopted ICT apps for its various services. Community pharmacies in the countries of the Organization for Economic Cooperation and Development, such as the United States and European countries, are already using ICT for various purposes, including for dispensing medicines, billing, and government reimbursements [4]. Furthermore, ICT apps are being used in community pharmacies to manage various services, including drug information systems, laboratory systems, logistics, and accounting [5]. A futuristic view shows that the adoption of ICT apps in the community pharmacy setting is expected to contribute to clinical decision making, achieving cost-effectiveness, expedited medication delivery times, and faster delivery of many other services [6].

The Malaysian health system is a dual-tiered system with a government-funded public sector and a private sector [7]. Pharmacists are an integral component of the Malaysian health system and play a vital role in regulatory control and policy work for community pharmacies, hospital and clinical pharmacy services, pharmaceutical production, academic activities, and health promotion [8]. Community pharmacists are the first-line health care providers for patients with minor ailments or those seeking health care services and over-the-counter medicines [9]. In Malaysia, community pharmacies operate as independent retail pharmacies, retail chain pharmacies, or pharmacies attached to a medical doctor’s clinic; they provide medicines, basic health care advice, and other pharmacy services such as health education and drug information.

Malaysia now has smart cities with integrated housing, marketplaces, public amenities, and transportation linkages that are facilitated and connected via ICT networks and apps [10]. To provide medicines and health services to the people living in these smart cities, community pharmacies need to reposition themselves and adopt ICT apps. Furthermore, the Malaysian community pharmacy sector faces challenges such as pressure to improve productivity and services to meet the needs of patients and consumers and the ever-increasing demand for various kinds of health services. The implementation of ICT in community pharmacies can streamline and help to overcome these challenges, especially for the effective delivery of pharmacy services [11]. However, there is a dearth of information regarding the utilization of ICT by community pharmacies in Malaysia. Therefore, the aim of the present study was to understand the positioning and utilization of ICT by community pharmacies in Malaysia, and to study its impact on the practices of community pharmacies toward realizing better productivity and health outcomes for patients.

## Methods

### Study Design and Setting

A cross-sectional study was conducted among community pharmacies using a self-administered questionnaire in the state of Selangor, Malaysia from November 2018 to January 2019.

### Participants

We obtained a list of the total number of pharmacies in the state of Selangor from the website of the Pharmaceutical Services Division, Ministry of Health, Malaysia. There were 1394 registered community pharmacies identified in the state of Selangor. We used Google Maps to locate pharmacies in 9 subdistricts and selected community pharmacies based on ease of access to obtain a minimum of 5 community pharmacies from each subdistrict. Accordingly, a total of 90 community pharmacies spread across 9 subdistricts of the state of Selangor were approached to participate in this study. In our study, the sampling unit was the community pharmacy. Consequently, we invited only one participant from pharmacies with more than one pharmacist.

### Study Instrument/Tool

A questionnaire was developed following a literature review on ICT use in the community pharmacy and health sector. We also obtained local literature on ICT and health in Malaysia to inform the development of the questionnaire [1-8,11]. The questionnaire comprised two parts. Part A encompassed the demographic characteristics of participants and their community pharmacies, and Part B encompassed the roles of ICT in community pharmacies. The questionnaire was finalized via a pilot study with 5 community pharmacists.

### Data Collection

The community pharmacies were approached by trained research assistants who invited the pharmacists to participate in the study. The questionnaire, along with a self-explanatory statement, was administered to the community pharmacies, and written consent to participate in the study was obtained. The participants were then asked to complete the questionnaire and were informed that they could complete it at their convenience and that the questionnaires would be collected after 1 week. The questionnaire was in English, and it was estimated to take 15 to 20 minutes to complete. After 1 week, the research assistants visited the pharmacies and collected the questionnaires. Owing to logistical barriers, no further follow-up visits were made.

### Data Analysis

The responses from the paper-based questionnaires were transferred into and analyzed using the Statistical Package for Social Sciences version 20.0 (SPSS Inc, Chicago, IL, USA). Categorical variables are presented as numbers and frequencies, whereas continuous variables are presented as means (SD). The association between sociodemographic characteristics and the adoption of ICT in community pharmacies was examined by the Chi square test and its alternative, Fisher exact test, when relevant. A *P* value of less than .05 was considered to indicate statistical significance.

### Ethics Approval

Ethics approval was obtained from the Monash University Human Research Ethics Committee (Project ID: 16602, date of approval October 1, 2018) prior to study commencement.

## Results

Among the 90 community pharmacies approached, 60 agreed to participate in the study, for a response rate of 67%. Among the respondents, the majority were female pharmacists, and more than half of the community pharmacies were run by young adults (ie, 30 years old and younger). Most of the respondents held a bachelor’s degree in pharmacy (Table 1). Regarding location and accessibility, most of the community pharmacies were located in residential areas, including towns and near markets. Some pharmacies were also located inside shopping malls and near hospitals. Most of the community pharmacies were independent retail pharmacies, followed by chain pharmacies. The majority of the community pharmacies remained open 8-12 hours per day. Community pharmacies provided a range of different services such as blood pressure measurement, blood glucose tests, blood cholesterol tests, and other services (eg, diet consultation, pregnancy tests, weight management, smoking cessation). The detailed results are summarized in Table 1.

As shown in Table 2, almost all of the community pharmacies were locatable via GPS and associated navigation apps such as Waze and Google Maps. Half of the community pharmacies used social media for the advertisement and promotion of their products. The majority of the community pharmacies were using electronic payment systems, including credit cards, and some were also using other new electronic payment methods such as Alipay, Boost, and Epay. Moreover, many of the community pharmacies were using software and programs for accounting and logistics purposes. In addition, the majority of participating community pharmacies were using a barcode reading system for medicines/health products. Overall, 16/60 (22%) of the pharmacies had online stores, and consumers could buy medicines and health products from these pharmacies via their online portal. In addition, a third of those pharmacies were using at least one of the common online business platforms in Southeast Asia to sell products/medicines (Table 2).

**Table 1:** Demographic and characteristic data of participants and their pharmacies (N=60).

Characteristics		n (%)^a^	
**Gender**			
	Male		24 (40)
	Female		36 (60)
**Age (years)**			
	21-30		32 (53)
	31-40		14 (23)
	41-50		9 (15)
	51-60		2 (3)
	61-70		2 (3)
	71-80		1 (2)
**Qualification**			
	Bachelor’s degree		38 (63)
	Master’s degree		8 (13)
	Diploma and other technical degree		14 (23)
**Location of community pharmacy**			
	In a shopping mall		7 (12)
	Near a residential area (eg, in a town, market)		44 (73)
	Near a hospital/clinic		7 (12)
	Rural area		2 (3)
**Type of community pharmacy**			
	Independent retail pharmacy		50 (83)
	Wholesale outlet		1 (2)
	Chain pharmacy		9 (15)
**Number of hours open**			
	8-12		53 (88)
	>12		7 (12)
**Number of staff members**			
	<5		32 (53)
	5-10		23 (38)
	>10		3 (5)
	Not reported		2 (3)
**Number of prescriptions per day**			
	5 or less		60 (100)
	>5		0 (0)
**Number of patients per day**			
	≤50		26 (43)
	50-100		15 (25)
	>100		5 (8)
	Not reported		14 (23)
**Number of medicines dispensed/sold per day**			
	≤50		20 (33)
	51-100		22 (37)
	>100		6 (10)
	Not reported		12 (20)
**Health services provided**			
	Blood pressure measurement		37 (62)
	Blood glucose test		34 (57)
	Blood lipid test		11 (18)
	Blood cholesterol test		25 (42)
	Blood uric acid		10 (17)
	Other services^b^		25 (42)

^a^Due to rounding, percentages may not add up to 100%.

^b^Other services include diet consultation, pregnancy tests, weight management, and smoking cessation.

Regarding automation and the use of technology in preparing and dispensing medicines, the majority of the community pharmacies (>90%) did not have a tablet-based or pill-counting machine or an automated unit-dose packing machine (Table 2). However, some community pharmacies had a labeling machine. As shown in Table 2, most pharmacies had computers with internet access. In addition, the majority of the pharmacies had an electronic record system and online accounts of the patients. The telephone was the most commonly used means of communication with patients, although some pharmacies also used email, WhatsApp, and text messaging.

With respect to the use of online resources to provide evidence-based medicine information, the majority of the community pharmacies had access to drug information resources online and through search engines. The pharmacies were using globally accessed medical and health-related portals such as Monthly Index of Medical Specialties, Medscape, National Pharmaceutical Regulatory Agency, and the British National Formulary to look at health- and medicine-related information (Table 2).

Statistical analysis was performed to examine whether there were any associations between the sociodemographic characteristics and the utilization of ICT in community pharmacies (Table 3). Several associations were noted. There was a statistically significant association between gender and clients having an online account in the pharmacy (*P*=.01). A total of 69% (25/36) of females reported having an online account compared with only 38% (9/24) of male respondents.

There were two statistically significant associations found among the associations evaluated: age of respondents was associated with having a labeling machine in the pharmacy and having mobile or online apps for the pharmacy store. Among pharmacists aged ≤30 years, 41% (13/32) reported having a labeling machine compared to only 14% (2/14) and 7% (1/14) of those aged 31-40 and >40, respectively. Similarly, 34% (11/32) of those aged ≤30 years reported having mobile or online apps for pharmacy stores compared to only 7% (2/28) of respondents aged above 30.

There was a statistically significant association between the type of pharmacy and the ability to receive patient information from different mobile health apps (*P*=.002), with 56% (5/9) of the chain pharmacies reporting receiving information compared to only 12% (6/50) of retail pharmacies. In addition, there was a significant association between the number of staff and having an online store for the pharmacy (*P*=.009) and receiving patient information from different mobile health apps (*P*=.01). In this study, 48% (11/23) of pharmacies with 5-10 staff members had an online store, compared to only 33% (1/3) and 13% (4/32) of pharmacies with more than 10 staff members and less than 5 staff members, respectively. In addition, 39% (9/23) of pharmacies with 5-10 staff members reported receiving patient information from different mobile health apps compared to only 6% (2/32) in pharmacies with less than 5 staff members.

**Table 2:** Utilization of information community technology in community pharmacies (N=60).

Variable		n (%)	
Locatable via GPS and other navigation systems			59 (98)
**Online advertisement medium**			
	Facebook		28 (47)
	Twitter		1 (2)
	Instagram		1 (2)
	None		30 (50)
Electronic payment systems			55 (92)
Tablet- or pill-counting machine			5 (8)
Barcode reading system for medicines/health products			47 (78)
Automated/unit-dose packaging machine			3 (5)
Labeling machine			16 (27)
Computer with internet facilities			56 (93)
Electronic patient record system			46 (77)
Online account of the clients			34 (57)
**Communication with regular clients**			
	Phone		50 (83)
	Email		12 (20)
	WhatsApp		9 (15)
	Text message		22 (37)
	Walk-in		26 (43)
	Facebook		1 (2)
	Fax		1 (2)
	No communication		1 (2)
Online store of the pharmacy			16 (27)
Mobile or online apps for pharmacy store			13 (22)
Receipt of patient information from different mobile health apps			11 (18)
Website/software for drug information			52 (87)
**Common website/software and sources for drug information**			
	MIMS^a^		41 (68)
	Google search		13 (22)
	Medscape		2 (3)
	National Pharmaceutical Regulatory Agency		3 (5)
	Micromedex, Lexicomp		1 (2)
	British National Formulary		1 (2)
	NHS^b^ Health		1 (2)
	Up-to-Date		1 (2)
	PubMed, NICE^c^		1 (2)
**Use of common online business platforms to sell products/medicines**			
	Lazada		10 (17)
	Shopee		6 (10)
	Esyms		8 (13)
	11street		2 (3)
	None		40 (67)
Software/programs for logistics and accounting			41 (68)

^a^MIMS: Monthly Index of Medical Specialties.

^b^NHS: National Health Service.

^c^NICE: National Institute for Health and Care Excellence.

**Table 3:** Associations between sociodemographic characteristics and adoption of information communication technology in community pharmacies.^a^

Variable	Gender	Age	Qualification	Location of pharmacy	Type of pharmacy	Number of staff
Locatable via GPS and other navigation systems	.40	.46	.90	.58	.65	.83
Online advertisement medium	.37	.02	.57	.17	.37	.86
Electronic payment systems	.99	.75	.44	.16	.62	.28
Tablet or pill-counting machine	.38	.15	.58	.95	.19	.33
Barcode reading system for medicines/health products	.31	.48	.19	.73	.58	.37
Automated/unit-dose packaging machine	.56	.40	.61	.53	.43	.67
Labeling machine	.27	.04	.80	.68	.39	.38
Computer with internet facilities	.67	.80	.64	.10	.13	.92
Electronic patient record system	.38	.29	.73	.29	.43	.18
Online account of the clients	.01	.13	.26	.53	.82	.16
Communication with regular clients	.44	.06	.10	.39	.12	.05
Online store of the pharmacy	.12	.32	.53	.90	.30	.009
Mobile or online apps for pharmacy store	.07	.04	.83	.74	.12	.05
Receipt of patient information from different mobile health apps	.68	.88	.74	.95	.005	.01
Website/software for drug information	.91	.71	.36	.34	.23	.80
Use of common online business platforms to sell products/medicines	.26	.69	.23	.61	.13	.52
Software/programs for logistics and accounting	.64	.58	.21	.40	.39	.31

^a^Values in the table are *P* values from the Chi square or Fisher exact test.

## Discussion

### Principal Findings

This study shows that community pharmacies in Selangor are partly utilizing ICT apps/services. The Malaysian urban landscape is changing with the increased use of ICT-driven integrated smart cities, where businesses and services are making use of ICT apps for effective delivery and functioning [2,10]. However, Malaysian community pharmacies are adopting ICT relatively slowly. This study shows that young community pharmacists who are computer savvy and who are active internet users are leading these changes, with a greater proportion of younger than older respondents reporting using mobile or online apps for their online pharmacy stores and adopting labeling machines (Table 3).

Most of the community pharmacies in this study are located in residential areas, towns, and markets, and provide greater access to medicines to people living in and around these areas. This is a different approach to community pharmacy positioning because earlier community pharmacies were mostly set up in a nearby colony near hospitals or medical clinics to provide easier access to consumers [12]. As people moved from traditional colonies of houses to modern condominiums with integrated housing and other facilities, community pharmacies needed to position themselves to serve their consumers better. However, the downside of the current positioning of community pharmacies might be that rural areas with sparse housing density may not have easier access to pharmacies. More rural clinics with pharmacies may have to be set up to bridge this gap of community pharmacies in rural areas [13].

Another aspect of ICT utilization is providing people with online access. Only some community pharmacies in our study had an online store, a mobile app, and products available via an online business platform such as Lazada and Shopee [14]. Unlike pharmacies, online trading platforms such as Lazada and Shopee are available for most of the other commercial products in Malaysia. Online pharmacies provide people with improved access, and they might provide them with cheaper medicines and the ability to save time and money, as consumers do not have to visit the community pharmacy in person [15]. However, regulating products in online pharmacies, especially health products such as cosmetics, nutraceuticals, and supplements imported from overseas, is a challenge, as these products might have different quality standards when they are manufactured in the host country. It is the responsibility of the regulating authorities to monitor online pharmacies, and the sales and distribution of medicines and health products via online business platforms [15,16]. In addition, privacy and security could be another concern with some forms of online purchasing, and there is some evidence suggesting that after the completion of consumers’ details and monetary transactions, the consumers could ultimately not receive the products they ordered, posing problems such as frustration and delay of care [16].

ICT can help community pharmacies integrate with the digital ecosystems of urban cities. This study showed the ability to find the location of community pharmacies via GPS and other smart navigation systems. This provision will help consumers, as well as visiting tourists and others, access community pharmacies more easily, especially in cities where people use internet-based apps and navigation systems to access businesses and services. A study by Watson et al [17] on the health-seeking behavior of consumers showed that convenience of location affects patients’ visits to community pharmacies for consultation for minor illnesses. The use of an ICT-based navigation system for locating pharmacies and the ability to check the availability of the necessary medicines will provide convenience as well as improved access to pharmacies for consumers.

ICT is an important means of communication and advertisement for different businesses and services [18]. This study showed that community pharmacies were using social media such as Facebook for advertisements, especially to improve the visibility and branding of pharmacy stores. A global study conducted by Benetoli et al [19] on participants from 8 countries (Australia, New Zealand, the United States, Brazil, Germany, Nigeria, Thailand, Philippines, and the United Kingdom) showed that social media positively impacted the pharmacy business by allowing community pharmacists to stay connected with customers or driving customers to their pharmacies [19]. Apart from advertisements, social media can be used for education and information dissemination, recruitment drives, and pharmacy services [19]. However, the dominant use of telephone, along with email, WhatsApp, and text messaging, shows that community pharmacies are diversifying the way they communicate and reach out to customers and patients. Staessen [20] reported that the use of reminders and follow ups with patients from community pharmacy staff via smartphone or text messaging services was effective in improving patients’ adherence to medicines.

Automation is a key development that is driving change in the community pharmacy sector globally [21]. This study shows the minimum use of automated devices in pharmacies, such as pill-counting machines, automatic fillers, and prescription scanners. However, few pharmacies used label-printing machines, and very few had pill-counting machines and unit-dose packaging machines. Nevertheless, there was widespread use of ICT such as software for logistics and accounting purposes by community pharmacies in this study. Studies have shown that the use of modern ICT apps such as electronic inventory systems and barcode technology leads to improved technical accuracy and a reduction in the incidence of potential medication dispensing errors [22,23]. Carmenates and Keith [24] showed that automation in community pharmacies will lead to time savings, thereby providing more time for pharmacists to focus on delivering pharmaceutical care to the patient. Moreover, the present study shows the widespread use of electronic payment systems, including credit cards and new methods of payment such as Alipay, Epay, and Boost by community pharmacies. This shows that Malaysian community pharmacies are adopting ICT apps for business and transactions, and are slowly preparing themselves for the next technological revolution.

Approximately half of the community pharmacies included in this study used electronic patient records. The electronic record system allows pharmacies to record detailed information about the patients. For instance, in Australia, community pharmacies utilized ICT tools such as My Health Record to keep data of patients in cloud storage systems to ensure better medication management [25]. The use of an electronic record system saves the pharmacists time in going through the hard files of patient medical records and makes it easier for community pharmacies to communicate with the general practitioner.

Our study shows that community pharmacies also used mobile apps to gain access to medical databases. Apidi et al [26] reported that Lexicomp, Epocrates, Micromedex, and Drugs.com were the most commonly used drug information sources. Registered pharmacists in the United Kingdom reported using mobile apps to access drug databases such as the British National Formulary and Martindale because of the simplicity, user friendliness, and up-to-date information offered [27]. However, the study also reported the role of factors such as risks, company policies, and lack of regulations that may hinder the use of mobile health apps in community pharmacies. Few community pharmacies in Malaysia have developed their own mobile apps to promote their products and services. The personal details of their consumers are recorded as members, and these members can enjoy the privilege of discounted prices. According to a study carried out by DiDonato et al [28], several factors prompt consumers to use mobile health apps, such as easy accessibility, privacy assurance, and beneficiaries, and consumers were less likely to use mobile apps when there were issues related to reliability, cost, and privacy. Mobile apps can help both the consumer/patient and community pharmacists. The greater challenge is to use mobile apps for patient self-management of diseases and to link the data from these mobile apps to pharmacies and health systems while creating an ecosystem that can regulate the use of these mobile health apps.

### Strengths and Limitations

This study provides useful data on the ICT and electronic health infrastructures in community pharmacies in the state of Selangor, Malaysia. However, some limitations of this study should be acknowledged. First, the study was performed with only 60 community pharmacies, as some declined to participate due to time constraints, especially community pharmacies with limited staff (ie, 1 or 2 staff members). Consequently, the study might not be representative of all pharmacies in Selangor and in Malaysia, which could affect the generalizability of the results, especially in rural areas. However, given the limited literature from Malaysia on this topic, we believe that the study findings are helpful in providing future guidance.

### Implications and Recommendations for Future Practice

We believe that more investment in ICT in the community pharmacy sector is needed in Malaysia. This approach would be aided by the good internet coverage in the state of Selangor and in Malaysia in general [29]. This could lead to several benefits to this sector. In particular, this could increase the business of community pharmacies, as clients and patients could have online access to all services and products, including online orders and delivery. It could also improve the population’s quality of life by freeing the pharmacists’ time to provide patient-centered services and counseling rather than focusing on time-consuming tasks than can be done perfectly by the technology. Additionally, it can help in reducing medication errors and other issues related to medication use. Furthermore, it can improve logistics and inventory management along with other managerial aspects of operating a pharmacy.

### Conclusion

Community pharmacies in Malaysia are using ICT apps and adapting to the pharmacy services needs of modern smart cities. However, the adoption of ICT apps for pharmacy services has been slow and varied. The use of ICT apps for accounting, logistics, and similar tasks was high, whereas the use of ICT for automation, dispensing, and pharmaceutical care service delivery was relatively low. Future community pharmacies will be driven by patient-centered services and the use of ICT apps. Further studies in Malaysia, with country-wide coverage, need to focus on ICT usage in community pharmacies, consumer preferences, and regulatory ecosystems that guide ICT usage in pharmacies.

## Abbreviations

ICT: information and communication technology
